# Fertility preservation training for obstetrics and gynecology fellows: a highly desired but non-standardized experience

**DOI:** 10.1186/s40738-017-0036-y

**Published:** 2017-07-04

**Authors:** Elizabeth J. N. Miller, Lisa M. Cookingham, Teresa K. Woodruff, Ginny L. Ryan, Karen M. Summers, Laxmi A. Kondapalli, Divya K. Shah

**Affiliations:** 1 Department of Obstetrics and Gynecology, University of Minnesota, 420 Delaware St SE, Minneapolis, MN 55455 USA; 2 Center for Reproductive Health, Kaiser Permanente Northern California, 1650 Response Road, Sacramento, CA 95815 USA; 3 Department of Obstetrics and Gynecology, Northwestern University, 303 East Superior Street, Chicago, IL 60611 USA; 4 Department of Obstetrics and Gynecology, Division of Reproductive Endocrinology and Infertility, University of Iowa, 200 Hawkins Drive, Iowa City, IA 52242 USA; 5 Colorado Center for Reproductive Medicine, 10290 RidgeGate Circle, Lone Tree, CO 80124 USA; 6 Department of Obstetrics and Gynecology, Division of Reproductive Endocrinology and Infertility, University of Pennsylvania, 3701 Market Street, Suite 800, Philadelphia, 19104 USA

**Keywords:** Fertility preservation, Fellowship training, Medical education

## Abstract

**Background:**

Despite a large body of data suggesting that delivery of fertility care to cancer patients is inconsistent and frequently insufficient, there is a paucity of literature examining training in fertility preservation for those physicians expected to discuss options or execute therapy. The study objective was to compare fertility preservation training between Reproductive Endocrinology & Infertility (REI) and Gynecologic Oncology (GYN ONC) fellows and assess the need for additional education in this field.

**Methods:**

A 38-item survey was administered to REI and GYN ONC fellows in the United states in April 2014. Survey items included**:** 1) Clinical exposure, perceived quality of training, and self-reported knowledge in fertility preservation; 2) an educational needs assessment of desire for additional training in fertility preservation.

**Results:**

Seventy-nine responses were received from 137 REI and 160 GYN ONC fellows (response rate 27%). REI fellows reported seeing significantly more fertility preservation patients and rated their training more favorably than GYN ONC fellows (48% of REI fellows versus 7% of GYN ONC fellows rated training as ‘excellent’, *p* < 0.001). A majority of all fellows felt discussing fertility preservation was ‘very important’ but fellows differed in self-reported ability to counsel patients, with 43% of REI fellows and only 4% of GYN ONC fellows able to counsel patients ‘all the time’ (*p* = 0.002). Seventy-six percent of all fellows felt more education in fertility preservation was required, and 91% felt it should be a required component of fellowship training.

**Conclusion:**

Significant variability exists in fertility preservation training for REI and GYN ONC fellows, with the greatest gap seen for GYN ONC fellows, both in perceived quality of fertility preservation training and number of fertility preservation patients seen. A majority of fellows in both disciplines support the idea of a standardized multi-disciplinary curriculum in fertility preservation.

**Electronic supplementary material:**

The online version of this article (doi:10.1186/s40738-017-0036-y) contains supplementary material, which is available to authorized users.

## Background

As survival rates among young cancer patients have continued to rise, the scope of cancer treatment has expanded to address long term survivorship and quality of life issues in individuals diagnosed with cancer [1, 2]. Young survivors consistently identify desire for future fertility as among the most significant concerns following a cancer diagnosis [1–4]. Heightened anxiety about fertility has been shown to negatively correlate with perceived quality of life among cancer survivors [5], whereas availability and uptake of fertility preservation services prior to treatment has been shown to increase psychosocial well-being and avoid long-term regret in patients diagnosed with cancer [6–10].

Though many fertility preservation techniques have existed for decades, it is only recently that the many medical specialties involved in cancer care have converged to start providing fertility options to young cancer patients. The term “oncofertility” was coined in 2006 to describe this integrated discipline that addresses the complex reproductive needs of cancer survivors by “balancing life-preserving treatments with fertility-preserving options [11–13]. Published guidelines on fertility preservation from the American Society of Clinical Oncology (ASCO) acknowledge the need for such a multidisciplinary approach, targeting a diverse array of specialists including medical oncologists, radiation oncologists, gynecologic oncologists, urologists, hematologists, pediatric oncologists, and surgeons [14]. Both ASCO as well as the American Society for Reproductive Medicine (ASRM) specify that all patients should be informed about the potential for infertility resulting from cancer and its treatment and given the opportunity to speak with a fertility specialist to discuss options for fertility preservation as early as possible [1].

Despite the clear desire for fertility preservation counseling on the part of both cancer patients and professional organizations, a large body of data suggests that delivery of fertility care to cancer patients is inconsistent and frequently insufficient [15–19]. Several barriers to referral for fertility preservation services have been identified, including the concern that fertility preservation procedures may compromise cancer care, the notion that pursuing fertility is not appropriate for a cancer patient who might not survive the disease, as well as discomfort and lack of knowledge about fertility management options [13, 20].

It is reasonable to assume that the self-perceived discomfort with discussion of fertility preservation options on the part of health care providers may stem from a lack of education in these techniques. There is a paucity of literature, however, specifically examining formal training in fertility preservation for those physicians expected to discuss options or execute therapy in practice. The objective of this study was to evaluate and compare the state of fertility preservation training for fellows in Reproductive Endocrinology & Infertility (REI) and Gynecologic Oncology (GYN ONC).

## Methods

We performed a cross-sectional mixed methods study of REI and GYN ONC fellows in 2014. The study included a 38-item online survey with a supplemental optional qualitative interview component. Only quantitative data from the survey component is presented in this report.

Fellowship coordinators for all REI and GYN ONC fellowship programs in the United States were contacted via e-mail and asked to distribute the survey link to their respective fellows. The survey link was sent twice between April and May of 2014. Data were collected using the Research Electronic Data Capture (REDCap) software (version 5.7.1) hosted at the University of Iowa [21]. Key survey measures to investigate FP training experience included exposure to FP, perceived quality and importance of FP training, ability to counsel patients on FP options, and need for more education in FP methods.

Specific survey questions are included in Additional file 1: Figure S1. The survey queried participant demographic information including fellowship type (REI versus GYN ONC), year of fellowship training (1, 2, 3, 4+), gender (female, male), age, and race (American Indian or Alaska native, Black or African American, Native Hawaiian or Other Pacific Islander, White, other, ‘prefer not to answer.’)

Participants were asked about the presence of a formal FP program at their fellowship institution (yes, no) and how many patients they see annually for FP counseling (>20, 10–19, < 10, none). To assess perceived quality and importance of FP training, participants were asked to rate the FP training in fellowship (excellent, good fair, poor) and to rate the importance of discussing FP options with patients (very important, somewhat important, neutral, rarely important, not at all important). Participants were asked if their current level of knowledge was adequate to counsel patients on FP options (‘yes, all of the time’, ‘yes, some of the time,’ ‘no’). To assess the need for additional training in FP, participants were asked if they were interested in an FP curriculum during fellowship (yes, no), the level of fellow participation (required, voluntary), and the desired format for a standardized curriculum (didactic lectures by faculty at their fellowship institution, a one-time intensive course at a centralized location, self-directed learning with online modules, other).

Descriptive analysis and frequencies were derived using REDCap software. Chi-square and Fisher’s exact tests were performed with SPSS (Version 22.0, Armonk NY) to compare demographics and key survey items between REI and GYN ONC fellows. These items included presence of formal FP programs, number of FP patients seen per year, quality of FP training, importance of discussing FP options with patients, ability to counsel FP patients, and self-reported need for more education in FP.

Due to low heterogeneity of response data for several survey questions, response categories were combined for the purpose of statistical analysis. Pertinent variables and new response categories include race (white or other), number of FP patients seen annually (> 20, 10–19, or <10), perceived quality of training in fellowship (excellent, good, or fair/poor), importance of discussing FP options with patients (very important or less than very important), and ability to counsel patients on FP options (all of the time or less than all of the time).

## Results

**Fig. 1:**
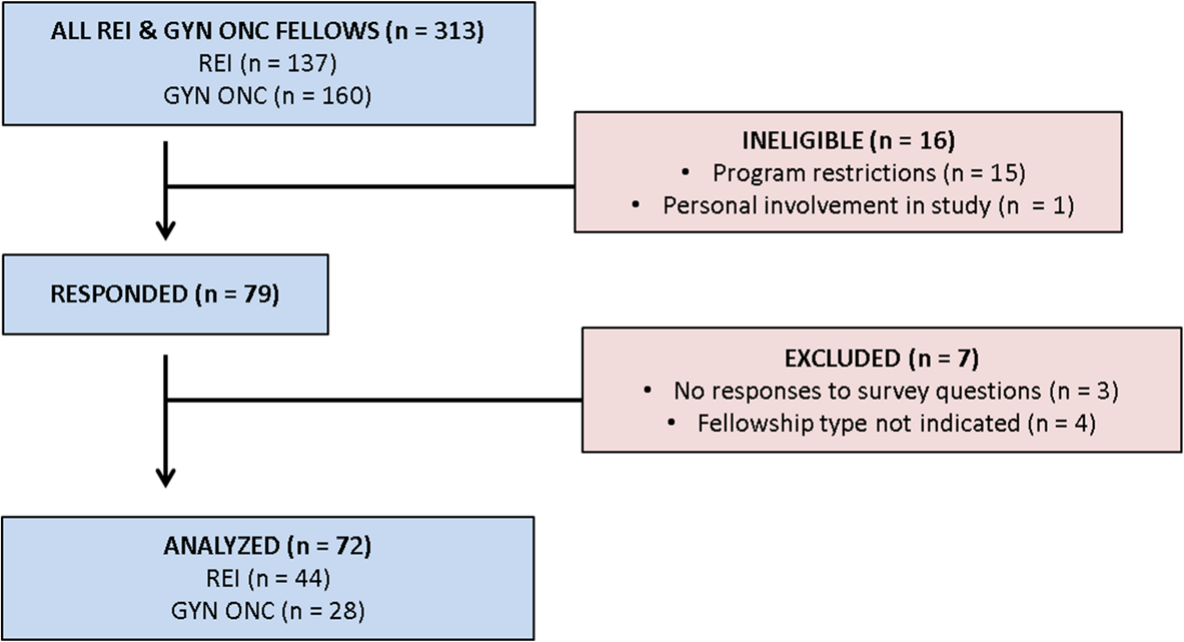
Flow diagram of study participants. Data from 72 of 79 total respondents were included in the study. *REI* = reproductive endocrinology and infertility, *GYN ONC* = gynecologic oncology

At the time of survey distribution, there were 146 REI fellows and 167 GYN ONC fellows enrolled in 42 and 46 accredited fellowship programs, respectively. A total of 9 REI fellows and 7 GYN ONC fellows were ineligible to participate due to program restriction on survey participation or personal involvement with the study. A total of 79 responses were received from the eligible 137 REI fellows and 160 GYN ONC fellows in training in the United States in 2014, for an overall response rate of 27%. Of the total responses, 7 were removed from analysis due to missing key information (3 were submitted without responses to any survey questions, and 4 were submitted without identification of fellowship type). The final 72 study participants included 44 REI fellows (response rate 32%) and 28 GYN ONC fellows (response rate 18%). Figure 1 shows the flow of study participants.

Characteristics of survey participants are detailed in Table 1. The majority of respondents were female (78%); this was consistent across both types of fellowship programs. The average age of participants was also similar for both REI (33.3 years, range 30–43 ys) and GYN ONC (32.8 ys, range 30–40 ys). Self-identified race was comparable among participants, and respondents were distributed consistently across year of training for all fellows. Fellowship training programs were distributed throughout the United States: 14% in the West, 33.8% in the Northeast, 32.4% in the South, and 19.7% in the Midwest.

**Table 1:** Characteristics of survey participants

	REI (*n* = 44)	GYN ONC (*n* = 28)	Total (*n* = 72)	*p-value*
Female gender	34 (77%)	22 (79%)	56 (78%)	*1.000*
Age	33.3 ± 2.7	32.8 ± 2.4	33.1 ± 2.6	*0.418*
White race	31 (78%)	23 (82%)	54 (79%)	*0.872*
Fellowship year				*0.943*
First year	14 (32%)	10 (36%)	24 (33%)	
Second year	15 (34%)	9 (32%)	24 (33%)	
Third year or greater	15 (34%)	9 (32%)	24 (33%)	
Annual FP patient volume				*0.004*
Greater than 20	18 (43%)	3 (12%)	21 (31%)	
10–19	14 (33%)	7 (27%)	21 (31%)	
Fewer than 10	10 (24%)	16 (62%)	26 (38%)	

Age is expressed as mean ± SD. All other categories are expressed as n (%). Comparisons are between REI and GYN ONC respondents

To investigate exposure to FP during fellowship, participants were asked about the presence of a formal FP program at their fellowship institutions and the number of FP patients seen per year. A majority of participating REI fellows (90%) indicated that there was a formal FP program at their training institution, while less than half of participating GYN ONC fellows (47%) identified such a program. There was a significant difference in the number of patients seen annually for FP, with REI fellows seeing more FP patients per year (*p* = 0.004), as detailed in Table 1. The number of FP patients seen per year did not differ significantly based on the participant’s year of fellowship training (*p* = 0.595, data not shown).

**Fig. 2:**
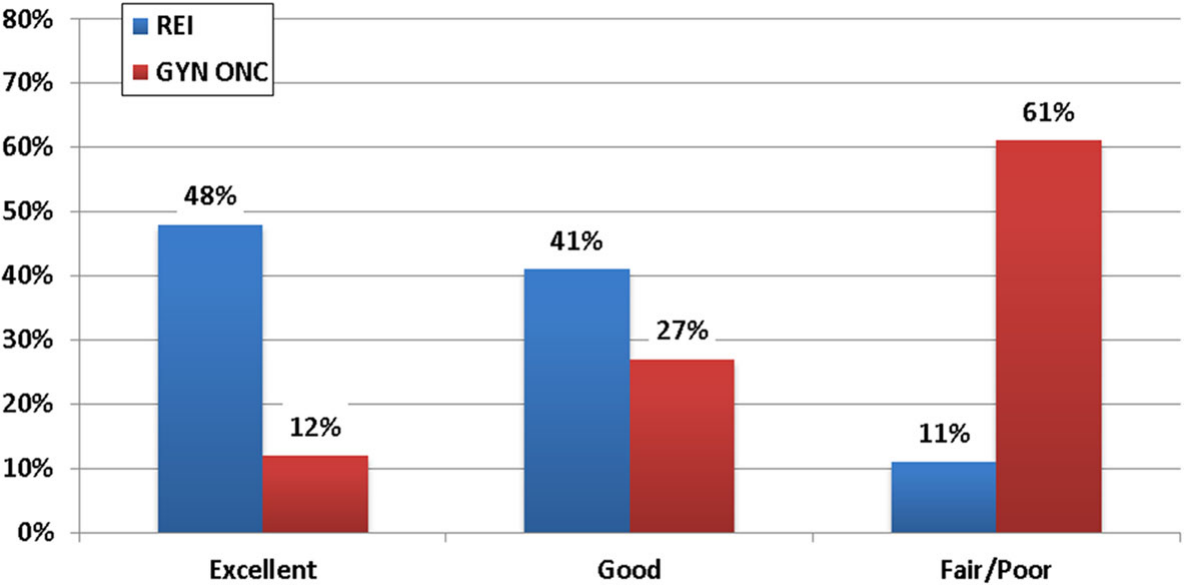
Perceived quality of fertility preservation training by fellowship type. Perceived quality of fertility preservation training varied significantly based on fellowship type. REI fellows rated training more favorably than GYN ONC fellows (Chi square test for trend = 17.377, *p* < 0.001)

**Fig. 3:**
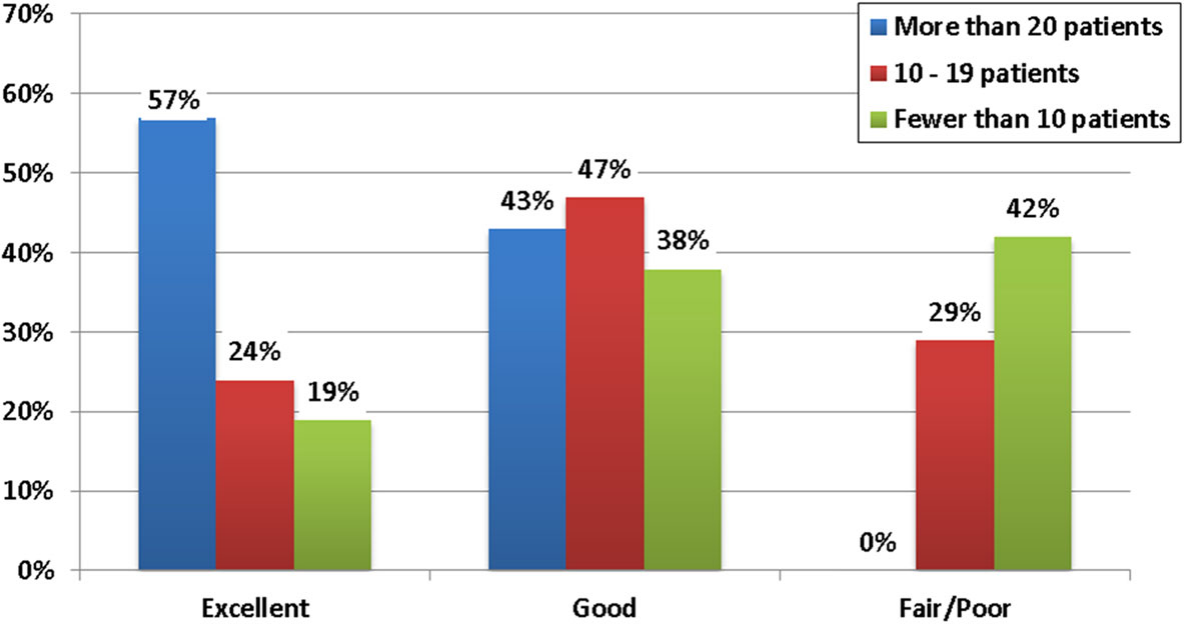
Perceived quality of fertility preservation training by volume of fertility preservation patients seen per year. Perceived quality of fertility preservation training varied significantly based on volume of fertility preservation patients seen (Chi square test for trend = 14.548, *p* = 0.006)

The perceived quality of FP training received during fellowship varied significantly based on fellowship type and number of FP patients seen annually. Specifically, REI fellows rated their training more favorably than GYN ONC fellows (*p* < 0.001), with 48% of REI fellows rating their training as ‘excellent’ and only 7% of GYN ONC fellows rating training as ‘excellent’ (Fig. 2). Fellows who see more patients annually also rated their training more favorably (*p* = 0.006, Fig. 3). The perceived quality of FP training did not differ significantly by year of fellowship (*p* = 0.281, data not shown).

Participants were asked about the importance of discussing FP options with patients and about their perceived ability to counsel patients based on their current level of knowledge. A majority of fellows (95% of REI fellows, 76% GYN ONC fellows) felt that discussing FP options with patients was ‘very important’. This response rate did not differ based on participant gender (*p* = 0.385, data not shown). There was a significant difference in self-reported ability to counsel patients on FP options based on subspecialty, with 43% of REI fellows and only 4% of GYN ONC fellows able to counsel patients ‘all the time’ (*p* = 0.002). The ability to counsel was also associated with the number of FP patients seen per year (*p* = 0.003). Of the participants who see greater than 20 FP patients per year, for example, 52% are able to counsel ‘all the time’ as compared to 30% of fellows who see 10–19 FP patients per year. Only 8% of fellows who see fewer than 10 FP patients per year are able to counsel ‘all the time.’ There was no significant difference in self-reported ability to counsel based on year of fellowship (*p* = 0.173).

A majority of all respondents (76%) felt they needed more education in FP, and 91% felt it should be a required component of fellowship training. Respondents were asked to identify their desired format for a hypothetical standardized educational curriculum in FP. Didactic lectures by faculty at their local institution received the highest endorsement (49%), followed by self-directed learning with online modules (24%), and a one-time intensive educational course at a centralized location (21%). A free response option was completed by four participants, and the following recommendations were made: “1-2 lectures on the basics of fertility preservation followed by a focused intensive exposure to caring for these patients,” “didactics along with practical experience,” “single overview lectures,” and “short rotation at a fertility preservation counselor clinic.”

## Discussion

This survey identifies substantial heterogeneity in fertility preservation training among REI and GYN ONC fellows in the United States. While the overwhelming majority of surveyed fellows felt that discussing fertility preservation options with patients was very important, only a minority of fellows in either REI or GYN ONC felt their current level of knowledge was adequate to consistently provide this counseling. A majority of fellows in both programs identified the need for more robust training in fertility preservation techniques including a desire for a standardized curriculum.

Results of this survey also identify a greater deficiency in fertility preservation training for GYN ONC as compared to REI fellows. REI fellows reported seeing a greater volume of fertility preservation patients than GYN ONC fellows, and this patient exposure was associated with both a higher perceived quality of training and an increased ability to counsel patients in fertility preservation options. While these findings are not surprising given the typical referral patterns of a newly diagnosed cancer patient from their oncology team to a REI for discussion of fertility preservation treatment, it is notable that only 4% of surveyed GYN ONC fellows felt that their level of knowledge was adequate to counsel patients regarding options for fertility preservation. This finding is particularly relevant for gynecologic oncology as compared to other medical oncology disciplines, as conservative surgery is often an integral component of fertility preservation for gynecologic malignancies.

While fertility preservation counseling and referral patterns have been extensively studied in the medical oncology literature, few studies have specifically examined this issue among gynecologic oncologists. A 2010 survey of 249 oncologists in the United States demonstrated that gynecologic oncologists were more likely than other oncologists to routinely consider fertility (95% versus 60%, *p* < 0.01) and consider modifying cancer treatment to preserve fertility (61% versus 37%, *p* < 0.01) [22]. When considering all oncologists together, referrals to reproductive endocrinologists were routinely made only 39% of the time, with 18% of oncologists never referring their patients. Other investigators have explored the factors associated with an oncology provider’s willingness to refer patients for fertility preservation counseling, and the implications of such referral patterns on patient well-being. Known barriers to referral include lack of knowledge or formal training in fertility preservation, lack of access to fertility preservation resources, and discomfort in discussing fertility in patients with a cancer diagnosis [20, 23]. The absence of coordinated oncologic and fertility care correlates with increased emotional distress among cancer patients [7]. Conversely, referrals for fertility care are more common among female physicians, physicians with favorable attitudes towards fertility preservation, and those whose patients routinely ask about fertility have to refer patients for fertility services [24]. This collaborative multidisciplinary approach is associated with increased patient satisfaction and ensures that adequate care is provided throughout the reproductive lifespan [7, 25].

A primary limitation of our study is the potential for selection bias with an overall response rate of 27%. It is possible that fellows who have an inherent interest and inclination towards fertility preservation were preferentially more willing to complete the survey. While this may result in our over-estimating fellow interest in fertility preservation training, it may also have caused us to under-estimate the true knowledge deficit among fellows. A second limitation is that of recall bias, as the number of fertility preservation patients seen annually is self-reported. Finally, there may also be important demographic differences between our participants and those in a larger national survey of all obstetrics and gynecology fellows in the United States [26]. While the average age of participants in our sample is similar, our study has greater percentages of female and white respondents, which may limit generalizability of the data.

Taken together, these findings illustrate the need for a coordinated multidisciplinary approach between oncologists and fertility specialists to optimize reproductive care for individuals diagnosed with cancer. This study not only demonstrates the desire for mandatory and standardized training in fertility preservation among REI and GYN ONC fellows, but also serves as an educational needs assessment to identify knowledge gaps as well as potential means by which they could be addressed. An easily accessible and cross-discipline training curriculum in fertility preservation is currently under development through a unique partnership between the American Society for Reproductive Medicine and the Oncofertility Consortium. This venture should not only help future health care providers acquire the requisite knowledge for patient counseling, but should also help promote collaboration and cross-specialty referrals in this multidisciplinary field.

## Conclusion

Significant variability exists in fertility preservation training for REI and GYN ONC fellows, with the greatest gap seen for GYN ONC fellows, both in perceived quality of fertility preservation training and number of fertility preservation patients seen. A majority of fellows in both disciplines support the idea of a standardized multi-disciplinary curriculum in fertility preservation.

## Additional file

Additional file 1: Figure S1. Survey instrument. The 38-item survey includes an assessment of demographic information, training, knowledge, and practice in fertility preservation, and an educational needs assessment for a standardized fertility preservation curriculum. (PDF 316 kb)

## Abbreviations

ASCO: American society of clinical oncology
ASRM: American society for reproductive medicine
GYN ONC: Gynecologic oncology
REI: Reproductive endocrinology and infertility
