# Supplementary material for: Fertility preservation training for obstetrics and gynecology fellows: a highly desired but non-standardized experience
Source: Fertil Res Pract. 2017 Jul 4;3:9. doi: 10.1186/s40738-017-0036-y (PMC5496430; doi:10.1186/s40738-017-0036-y)
Supplement: Additional file 1: Figure S1. — Survey instrument. The 38-item survey includes an assessment of demographic information, training, knowledge, and practice in fertility preservation, and an educational needs assessment for a standardized fertility preservation curriculum. (PDF 316 kb) [file 40738_2017_36_MOESM1_ESM.pdf]

Section 1 – Personal Demographics

1. Gender
    a. Female
    b. Male
2. Current age: ____

3. Race (mark all that apply)
    ___ American Indian or Alaska native
    ___ Asian
    ___ Black or African American
    ___ Native Hawaiian or Other Pacific Islander
    ___ White (non-Hispanic or Hispanic)
    ___ Other
    ___ I'd prefer not to answer

4. Have you ever been diagnosed with cancer?
    a. No
    b. Yes
        Type of cancer: ________
        Are you currently receiving treatment (medical or surgical?)
            a. No
            b. Yes
        Are you currently in remission?
            a. No
            b. Yes
5. Has your mother ever been diagnosed with cancer?
    a. No
    b. Yes
        Type of cancer: _________
6. Has your father ever been diagnosed with cancer?
    a. No
    b. Yes
        Type of cancer: _________
7. Have any of your siblings ever been diagnosed with cancer?
    a. No
    b. Yes
        Type of cancer: _________
8. Has your partner/husband/wife ever been diagnosed with cancer?
    a. No
    b. Yes
        Type of cancer: _________
9. Have any of your children ever been diagnosed with cancer?
    a. No
    b. Yes
        Type of cancer: _________

10. Have you ever been the primary caregiver to someone diagnosed with cancer?
  a. No
  b. Yes
    Relationship: ________
    Type of cancer: ________

Section 2 – Training Demographics

1. Fellowship training
  a. Reproductive Endocrinology and Infertility (REI)
  b. Gynecologic Oncology (GYN ONC)

2. Current year of fellowship training
  a. Frist year
  b. Second year
  c. Third year
  d. Fourth year or greater

3. Location of your fellowship program (by state): ________

4. Location of your residency program (by state): ________

5. What type of practice do you plan to pursue following fellowship?
  a. Academic/university hospital or clinic
  b. Private/ community hospital or clinic
  c. Hybrid of academic/university and private/community
  d. Research only (no clinical practice)
  e. Unsure

6. Have you heard of the field of fertility preservation or oncofertility?
  a. No
  b. Yes

Section 3 – Assessment of Training

1. Have you received any training in the listed treatment options (specifically for fertility preservation) during fellowship? (mark all that apply)
  ___ Didactic lectures
  ___ Fertility preservation consults with attending
  ___ Informal discussion with attending
  ___ Wet lab for surgical techniques
  ___ During live surgery
  ___ Other: ________

2. Did you receive any training in the listed treatment options (specifically for fertility preservation) during residency? (mark all that apply)
  ___ Didactic lectures

___ Fertility preservation consults with attending
___ Informal discussion with attending
___ Wet lab for surgical techniques
___ During live surgery
___ Other: ________

3. Where else have you learned about fertility preservation options? (mark all that apply)
___ Medical journals
___ Websites from academic institutions
___ Websites for the general public
___ Post-graduate course at medical conference
___ National guidelines
___ Other: ________

4. How would you rate the quality of your training in fertility preservation options during fellowship?
a. Excellent
b. Good
c. Fair
d. Poor

5. How would you rate the quality of your training in fertility preservation options during residency?
a. Excellent
b. Good
c. Fair
d. Poor

6. Have you received training on the following fertility treatment options? (mark all that apply)
___ In vitro fertilization with embryo cryopreservation
___ Oocyte cryopreservation
___ Ovarian tissue cryopreservation
___ Ovarian suppression with GnRH agonists prior to treatment
___ Ovarian transposition
___ Radical trachelectomy
___ Sperm cryopreservation
___ Testicular tissue cryopreservation
___ Radiation shielding

7. Does your fellowship institution have a formal fertility preservation program?
a. Yes
b. No
c. I don't know

8. Did your residency institution have a formal fertility preservation program?
   a. Yes
   b. No
   c. I don't know

Section 4 – Assessment of Current Knowledge and Practice

1. Do you currently see (or have seen) patients for fertility preservation in your fellowship program?
   a. Yes, I typically see > 20 patients per year seeking fertility preservation treatment options
   b. Yes, I typically see 10-19 patients per year seeking fertility preservation treatment options
   c. Yes, I typically see < 10 patients per year seeking fertility preservation treatment options
   d. No, I have never seen a patient seeking fertility preservation treatment options

2. Of the patients you see (or have seen) for fertility preservation, what percentage are seeking treatment due to a diagnosis of CANCER? ______(%)

3. Of the patients you see (or have seen) for fertility preservation, what percentage are seeking treatment due to a diagnosis (OTHER THAN CANCER) requiring therapy that could affect the patient's fertility (i.e. chemotherapy, radiation, surgery)? ______(%)

4. Of the patients you see (or have seen) for fertility preservation, what percentage are seeking treatment due to a desire to bank gametes or embryos for future infertility? ______(%)

5. How would you rate your current level of knowledge about the following fertility preservation options (Not at all knowledgeable/Aware but not very knowledgeable/ Knowledgeable/Very knowledgeable)
   a. Oocyte cryopreservation: ________
   b. Ovarian tissue cryopreservation: ________
   c. Ovarian suppression with GnRH agonists prior to treatment: ________
   d. Ovarian transposition: ________
   e. Radical trachelectomy: ________
   f. Sperm cryopreservation: ________
   g. Testicular tissue cryopreservation: ________
   h. Radiation shielding: ________
   i. Options for pre-pubescent females: ________
   j. Options for pre-pubescent males: ________

6. Do you feel your current level of knowledge is adequate to counsel patients about their fertility preservation options?
   a. No

b. Yes, some of the time
c. Yes, all of the time

7. How important is discussing fertility preservation options with patients?
a. Very important
b. Somewhat important
c. Neutral
d. Rarely important
e. Not important

8. Please check which of these fertility preservation therapies you have observed and/or performed
___ In vitro fertilization with embryo cryopreservation
___ Oocyte cryopreservation
___ Ovarian tissue cryopreservation
___ Ovarian suppression with GnRH agonists prior to treatment
___ Ovarian transposition
___ Radical trachelectomy
___ Sperm cryopreservation
___ Testicular tissue cryopreservation
___ Radiation shielding

9. When unable to provide a specific fertility preservation therapy to your patient, how likely are you to refer them to the appropriate consultant?
a. Very likely – I make (or have made) referrals on a consistent basis
b. Somewhat likely – I try to make (or have made) referrals, but on an inconsistent basis
c. Not likely – the following issues are barriers to referral (mark all that apply)
___ Lack of time during the visit
___ My limited knowledge of fertility preservation options
___ I don't feel comfortable discussing fertility preservation for moral, ethical, or religious reasons
___ Lack of services available in my region to offer fertility preservation options
___ Poor success rates of available fertility preservation options
___ My patients are too ill to undergo fertility preservation therapy
___ The prognosis of my patients is too poor to undergo fertility preservation therapy
___ My patients cannot afford fertility preservation therapy
___ I don't think my patients are interested in fertility preservation options
___ Other: _________

Section 5 – Educational Needs Assessment

1. Do you feel that fertility preservation training is necessary in your fellowship?

a. No, I do not think it is necessary
b. Yes, I think it is necessary, but only on a voluntary basis
c. Yes, I think it is necessary and should be part of the required curriculum

2. Do you feel that you need more education in fertility preservation therapies or techniques?
   a. No, I do not feel that I need more education
   b. Yes, I feel that I need more education

3. Would you be interested in a curriculum on fertility preservation therapies or techniques during your fellowship?
   a. Yes, I would be interested in a REQUIRED curriculum on fertility preservation therapies or techniques
   b. Yes, I would be interested in a VOLUNTARY curriculum on fertility preservation therapies or techniques
   c. No, I am not interested in a required or voluntary curriculum on fertility preservation therapies or techniques

4. If a standardized educational curriculum in fertility preservation were to exist, what format do you believe would be the most beneficial?
   a. Didactic lectures by faculty at my training program
   b. One-time intensive educational course at a centralized location
   c. Self-directed learning with online modules
   d. Other: ________

5. What do you feel will be the 3 most significant obstacles to adopting fertility preservation in your future practice?
   ___ Lack of time during the visit
   ___ My limited knowledge of fertility preservation options
   ___ I don't feel comfortable discussing fertility preservation for moral, ethical, or religious reasons
   ___ Lack of services available in my region to offer fertility preservation options
   ___ Poor success rate of available fertility preservation options
   ___ My patients are too ill to undergo fertility preservation therapy
   ___ The prognosis of my patients is too poor to undergo fertility preservation therapy
   ___ My patients cannot afford fertility preservation therapy
   ___ I don't think my patients are interested in fertility preservation options
   ___ I'm not interested in discussion fertility preservation options because I don't feel it is important
   ___ Other: ________
